# Using Coronary Artery Calcification Combined with Pretest Clinical Risk Assessment as a Means of Determining Investigation and Treatment in Patients Presenting with Chest Pain in a Rural Setting

**DOI:** 10.1155/2015/582590

**Published:** 2015-02-05

**Authors:** Baskar Sekar, Mark Payne, Azad Hanna, Abdul Azzu, Martin Pike, Michael Rees

**Affiliations:** ^1^Department of Cardiology, Ysbyty Gwynedd, Bangor LL57 2PW, UK; ^2^School of Medical Sciences, Bangor University, Brigantia Building, Penrallt Road, Bangor LL57 2DG, UK; ^3^Department of Radiology, Ysbyty Gwynedd, Bangor LL57 2PW, UK

## Abstract

462 patients presenting with chest pain to a rural district general hospital underwent calcium scoring and pretest clinical risk assessment in order to stratify subsequent investigations and treatment was retrospectively reviewed. The patients were followed up for two years and further investigations and outcomes recorded. Of the 206 patients with zero calcium score, 132 patients were immediately discharged from cardiac follow-up with no further investigation on the basis of their calcium score, low pretest risk of coronary artery disease, and no significant incidental findings. After further tests, 267 patients were discharged with no further cardiac therapy, 88 patients were discharged with additional medical therapy, and 19 patients underwent coronary artery by-pass grafting or percutaneous intervention. 164 patients with incidental findings on the chest CT (computed tomography) accompanying calcium scoring were reviewed, of which 88 patients underwent further tests and follow-up for noncardiac causes of chest pain. The correlations between all major risk factors and calcium scores were weak except for a combination of diabetes and hypertension in the male gender (*P* = 0.012), The use of calcium scoring and pretest risk appeared to reduce the number of unnecessary cardiac investigations in our patients: however, the calcium scoring test produced a high number of incidental findings on the associated CT scans.

## 1. Introduction

Coronary artery calcification has been used for a considerable amount of time as an assessment of risk for coronary artery disease. In the UK, the coronary calcium score has been incorporated into guidelines produced by the National Institute of Clinical Excellence (NICE), which has in turn developed an algorithm for the use of imaging investigations into chest pain of recent onset [1]. This guidance was developed by analysis of the currently available evidence on the use of imaging in the management of coronary artery disease. One specific recommendation is that exercise testing should no longer be used in the UK as a means of determining the probability of coronary artery disease.

Coronary calcification has been noted as an incidental finding on chest computed tomography for some time [2]; however, a large number of studies have demonstrated both prospectively and retrospectively that coronary calcification is a sensitive indicator of risk of adverse cardiac events and this has led to the publication of joint guidelines of the use of computed tomography in chest pain by the European Society of Cardiac Radiology and the North American Society for Cardiac Imaging [3].

More recently there has been the development of guidelines on the use of other imaging modalities which can follow on from the detection of coronary calcium, such as the use of single photon emission computed tomography (SPECT) in myocardial imaging [4]; these new guidelines have been reflected in both the updated NICE guidance and the practice in most hospitals in the UK. Among the most compelling data is the value of a zero calcium score which has been outlined in a number of papers and was also a feature of a paper written by the European Society of Cardiac Radiology [5]. Whilst the early work on calcium scoring concentrated on the risk of presence of coronary artery disease in symptomatic patients, there is continuing debate of the value of this test for screening for coronary artery disease, the value of which is being increasingly doubted [6].

A question remains as to whether calcium scoring should be the first line investigation in symptomatic patients or whether patients should go straight to computed tomographic coronary angiography (CTCA) or invasive coronary angiography; the case for using calcium scoring as a first line investigation has been made in the UK NICE [1] guidance and supported by many authors [7–9], some of which argue that using calcium scoring as a first line diagnosis rather than CTCA may be more cost effective [10]. This approach may also be very useful in populations where access to CTCA is limited. Others point to its wide applicability with very few exclusions [11]. Certainly calcium scoring performed without CTCA is a quicker technique with a lower radiation dose [12] and very much quicker than an exercise ECG; a typical calcium score takes approximately 5 minutes to perform and be interpreted [13]. Combining calcium scoring and CTCA results in a higher radiation dose and a longer examination. The question as to the role of calcium scoring as the preliminary step of cardiac imaging investigation should therefore be further explored.

In order to investigate whether the use of calcium scoring alone as a first line test is helpful in the diagnosis of chest pain, we undertook a prospective audit of 463 patients who undertook this test as the primary preliminary test for coronary artery disease and followed these patients up for a period of two years.

## 2. Methods

### 2.1. Patient Population

All patients assessed were from a population in North West Wales with a hospital catchment area of approximately 250,000 patients. All patients with stable chest pain attending chest pain or associated medical clinics were entered into the study.

The patient data, which was collected, included age, gender, cholesterol and lipid levels, and the presence of hypertension and diabetes. Outcomes measured included the number of coronary angiograms performed and their result, the number and type of other tests performed, and the presence of incidental findings. Patients were followed up for two years in the cardiology clinic or by rereferral or representation in the case of patients who had been discharged.

### 2.2. Scan and Postprocessing Protocol

Calcium scoring was carried out on a Symbia T16 Scanner. Following patient preparation and attachment of ECG leads, the patient was placed feet first into the scanner in a supine position and an anteroposterior and lateral topogram was acquired at 25 milliamperes (mA) and 130 kilovolts (kV) with a 5.9-second scan time, using a 0.6 mm slice thickness. The trigger and scan area for the calcium scoring scan was then set and the calcium scoring scan carried out with a 0.5-second rotation time at 37 mA and 130 kV with a pitch factor of 0.31 using retrospective ECG triggering. Slice thickness was set at 2.4 mm. All scans were acquired using a Siemens Caredose protocol giving an average exposure for males of 2.25 millisieverts (mSv) and for females 2.68 (mSv).

The calcium score is then calculated using an Agatston scoring system by examination of the regions of interest using a Symbia IQSPECT workstation with a computed tomography work package using Syngo protocols. Following this, the chest CT component was examined on a Fuji patient archiving computer system (PACS) and reported for incidental findings by experienced radiologists; all incidental findings on the CT scans were recorded.

A cut-off for the maximum calcium score was determined to be 1500. All calcium scores above this level were classified as a calcium score of 1500. Calcium scores above zero but less than 1 were scored as a calcium score of 1.

Calcium score results were analysed in relation to age, gender, and pretest probability. The analysis of risk factors and test results were also stratified according to age and gender.

### 2.3. Data Analysis

Data was recorded on a Microsoft Excel worksheet and analysed using SPSS 22 (IBM). Patient gender, age, pretest, risk and calcium score data was initially explored using the descriptive statistics package in SPSS to calculate means and distribution. Pearson correlation coefficients were calculated for the relationship between the main data variables. *t*-tests and a one-way ANOVA (analysis of variance) were used to compare variables. Continuous variables were expressed as mean ± standard deviation. Categorical variables were expressed as percentages.

## 3. Results

### 3.1. Patient Population

462 patients were entered into the study (age range 29–85, mean 58.7 ± SD 10.5 years); of these 259 were female (age 29–85, mean 59.7 ± SD 10.6 years) and 203 male (age 30–84, mean 57.4 ± SD 10.32 years). Patients were assessed as having a pretest probability of coronary disease according to the methodology outlined in NICE guidance [14]. These levels of risk were then simplified to three categories of risk: (a) low risk (0–29%), (b) medium risk (30–60%), or (c) high risk (>60%).

The mean pretest probability for coronary artery disease for this group was (a) female 36.56 ± SD 27.4 (medium pretest probability) and (b) male 65.66 ± SD 26.8 (high pretest probability).

### 3.2. Calcium Score (Figures 1, 2, and 3)

**Figure 1:**
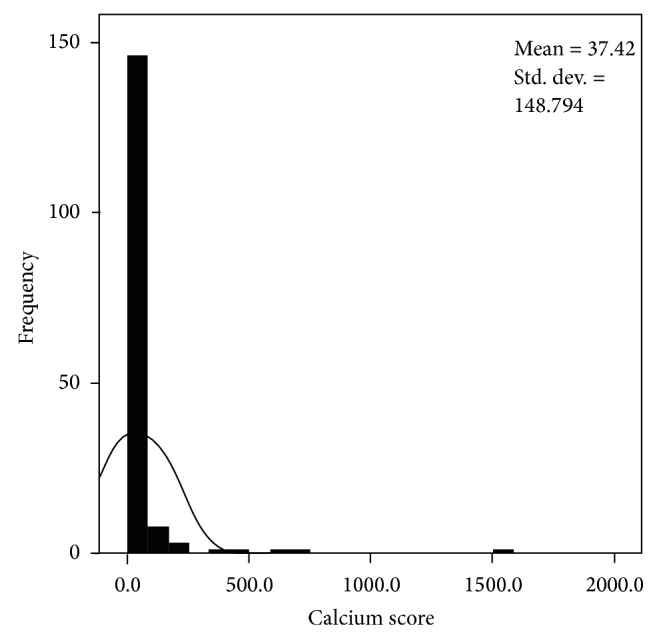
Distribution of calcium scores in patients with a low pretest risk of coronary disease.

**Figure 2:**
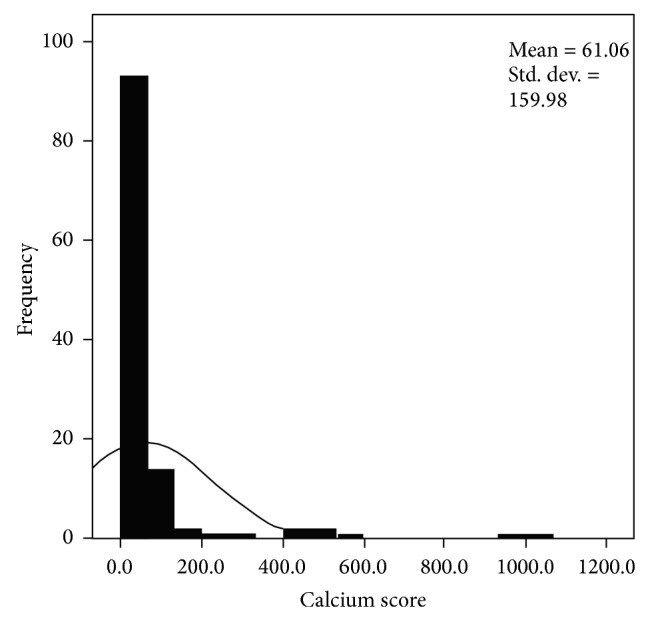
Distribution of calcium scores in patients with a medium pretest risk of coronary disease.

**Figure 3:**
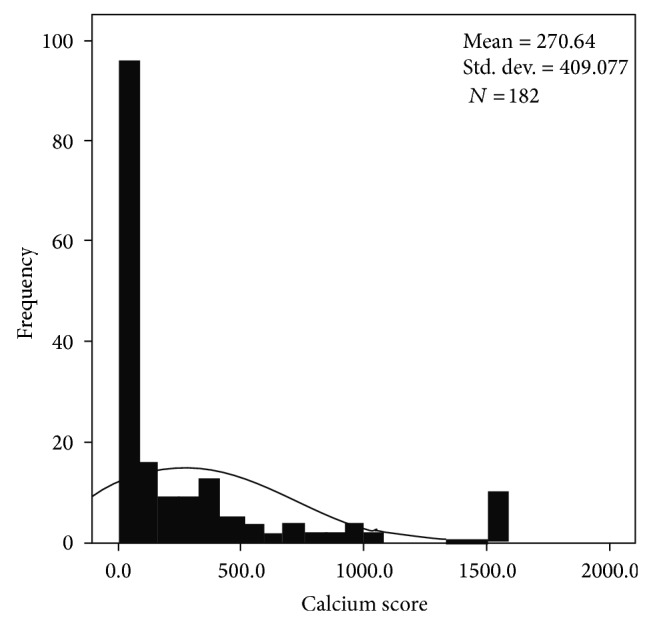
Distribution of calcium scores in patients with a high pretest risk of coronary disease.

The distribution of calcium scores is shown in Figures 1–3. This demonstrates that a zero calcium score was the commonest finding and that a zero calcium score may be found in all age groups and all risk groups.

The mean calcium score was calculated as follows: female 90.94 ± SD 245.6 and male 191.96 ± SD 356.4.

### 3.3. Relationship between Calcium Score, Diabetes, and Hypertension (Table 1)

Analysis of the relationship between diabetes, hypertension, and calcium score showed little relationship between these factors, except between calcium score and a combination of diabetes and hypertension for the male gender (*P* = 0.012). The other relationships showed either no significant difference or very weak relationships. The findings are summarized in Table 1.

### 3.4. Relationship between Pretest Risk and Calcium Score (Table 2)

132 patients had a low pretest risk and a zero calcium score; the majority of these were female patients (*n* = 114). 72 patients had a zero calcium score and medium pretest risk (female *n* = 52) and 50 patients had a zero calcium score and a high pretest risk (female *n* = 29). The results are summarised in Figures 1, 2, and 3 and Table 2.

The Pearson correlation (*r*) for pretest probability and calcium score for gender was 0.322 for male patients and 0.366 for female patients. Both have statistical significance to the 0.01 level (Table 3).

The overall correlation between pretest probability of coronary disease and calcium score gave a Pearson correlation (*r*) of 0.491, which is equivalent to 0.01 level indicating statistical significance. The correlation between calcium score and age gave a Pearson correlation of *r* = 0.340, which is also significant to the 0.01 level with statistical significance.

### 3.5. Relationship between Coronary Angiogram Findings, Pretest Risk, and Calcium Score (Table 3)

Coronary angiograms were performed on 53 male patients and 32 female patients; the findings are summarized in Table 3.

All the angiograms with moderate or significant findings had calcium scores above 10. Two patients with significant coronary artery disease had calcium scores in the range 10–100. No patients with a zero calcium score had a positive angiogram; one patient with a calcium score of 6 had a 30% stenosis in the left anterior descending coronary artery, and this patient had a high pretest probability. Thirty patients with a calcium score above 400 had positive angiograms with only 5 patients with a calcium score above 400 having negative angiograms.

### 3.6. Relationship between Cholesterol and HDL Levels and Calcium Score

There was no significant relationship between calcium score and cholesterol level.

### 3.7. Relationship between Calcium Score and Follow-Up Cardiac Investigations

71 female patients underwent exercise tests of which 37 were inconclusive (mean calcium score 52.86) and 32 negative (mean calcium score 35.5); one test was positive (calcium score 38). This patient had a subsequent normal coronary angiogram. A further test precipitated a broad complex tachycardia (calcium score 0). Sixty-six male patients had exercise tests of which 35 were negative (mean calcium score 68.8) and 31 inconclusive (mean calcium score 192.2). One patient with a negative exercise test had moderate coronary artery disease on angiography, while 2 patients with inconclusive exercise tests had moderate coronary artery disease on angiography; 4 patients with inconclusive tests had normal coronary angiograms.

Seven female patients had myocardial perfusion scans (MPI), all of which were negative. Four male patients underwent MPI scans, all of which were negative.

### 3.8. Incidental Findings and Resulting in Further Follow-Up Investigations

134 patients with a zero calcium score had no incidental findings on their computed tomography scans; 65 patients with a zero calcium score had incidental findings on computed tomography.

In total 155 patients (87 female) had incidental findings on the computed tomography scans; most of these were considered nonsignificant requiring no further action. The more significant findings included 18 patients with lung nodules or lymph nodes, requiring further assessment, 7 patients with ground glass pulmonary shadowing, and 9 patients with significant pleural-based abnormalities including 2 patients with pleural effusion and two patients with calcified plaques and one patient with a pleural-based mass. 16 patients had a hiatus hernia, which may have contributed to their symptoms. One patient had possible tuberculosis; one patient had a possible liver metastasis, which was found to be a simple cyst on follow-up. Further 6 patients were found to have liver cysts.

### 3.9. Patient Outcomes (Table 4)

Of the 267 patients who were discharged from follow-up cardiac care at the hospital with no further cardiac treatment, 132 patients were discharged on the basis of having a zero calcium score and low pretest probability. The remaining 135 were discharged from cardiac care after negative subsequent tests and follow-up consultation with no change in therapy.

Of the remaining 195 patients, 88 were given further medical treatment and advice, which included primary and secondary prevention. 19 patients underwent revascularisation by either percutaneous coronary angioplasty or coronary by-pass grafting, the revascularisation group representing 4.1% of the total patients number. The remaining 88 patients are undergoing additional tests and follow-up which include investigation of incidental findings on the chest CT, which formed part of the calcium scoring investigation. Other cardiac syndromes, which were diagnosed in follow-up, included paroxysmal atrial fibrillation, hypertrophic cardiomyopathy, and aortic stenosis.

Four patients with zero calcium scores presented again with chest pain but no evidence of troponin rise. After subsequent investigations, no evidence of coronary disease was found. Five patients with significantly raised calcium scores experienced an acute cardiac event, and one of these was fatal.

## 4. Discussion

There are still centres that do not have access to high quality, high speed cardiac computed tomographic imaging. It is generally accepted that a minimum specification of 64-slice CT is required for good cardiac diagnosis of coronary artery disease [1]. Some centres will still have only 4- or 16-slice CT available, which can perform calcium scoring but not CTCA. Even when CTCA is available, a strategy for using calcium scoring as the initial investigation may be more effective than routine use of CTCA as the first line investigation.

All the patients in this study were symptomatic but with stable symptoms and presented either to chest pain or other cardiac clinics. Of the 462 patients investigated, 132 patients could be immediately reassured that their combined risk assessment and calcium score meant that they could be discharged without further cardiac investigation or treatment; however, some of these patients required further follow-up for incidental findings on their CT scans.

Current guidance suggests [1] that patients with a calcium score of above 1 and below 400 should have CTCA and that patients with calcium scores above 400 should be considered either for a stress imaging test if the risk assessment is moderate, but if the risk assessment is high, they should undergo angiography. In our patient group, 157 patients would have been suitable under UK guidelines to have CTCA were this available. In this group of patients, only 10 underwent coronary intervention or surgery after coronary angiography. It could be argued therefore that had CTCA been used in this group, approximately 25% of the invasive coronary angiography examinations which were subsequently proved to be normal could have been avoided. We also performed calcium score examinations on 172 patients with high pretest risk; of these patients, 50 had a zero calcium score, indicating that pretest risk by itself may not effectively stratify patients with chest pain.

The combination of assessment of pretest risk and the use of calcium score is arguably a very strong rationale for the determination of subsequent cardiac investigation in patients with stable chest pain. Patients with a combination of zero calcium scores and low pretest risk should not require further cardiac investigations or treatment for coronary artery disease. Our study shows a moderately strong level of correlation between these two assessments (*P* = 0.01).

Previous large studies have shown the importance of the relationship between CTCA and pretest probability (CORE-64 Study) [15] in the investigation of patients with chest pain rather than relying on CTCA alone. This latter study also demonstrated that high calcium in the coronary artery reduced the accuracy of CTCA which indicates that a CTCA strategy alone may be inaccurate in these patients. A recent study on patients with zero calcium scores using positron emission tomography has shown that 16% of patients with a zero calcium score have inducible ischaemia [16]. In our series, no patients with a zero calcium score had positive angiography and only one patient with a low calcium score had a positive angiogram demonstrating a moderate lesion (30% left anterior descending coronary artery); this patient had a high pretest probability of coronary disease. Many studies have shown that significant coronary stenoses can occur in patients with low calcium scores [17], whilst some studies found positive angiograms in patients with zero calcium scores [18]. One such study was a subset of the CORE 64 study consisting of 71 patients with a zero calcium score who were examined and found to have a 12.5% requirement for revascularisation [19]. This finding together with our study would argue against the use of calcium scoring as the sole determinant of further investigation. Pretest probability is an established method of determining the risk of coronary artery disease [20] and has been used in most trials of noninvasive imaging for coronary artery disease [21]. It has been proposed that not only should pretest probability not be discarded in favour of using calcium scoring but the combination of the two methods is a more powerful predictor of the need for further investigation and treatment. The CORE 64 study also concluded that both calcium scoring and pretest probability were important for the effectiveness of CTA to confirm or exclude the presence of obstructive coronary artery disease.

It is of interest to note that our study showed either weak or nonsignificant relationships between various risk factors and calcium score. There was no relationship between cholesterol or lipid levels and calcium scoring. The strongest relationship was between a combination of diabetes and calcium scoring for the male gender and a lesser relationship between diabetes and the female gender.

Our study also demonstrated that when the whole of the CT examination was examined, numerous other findings were found. The adjunct CT was reported by an experienced radiologist in all cases and a very large number of these “incidental” findings were recorded. Most of these findings were judged to be benign and of no significant clinical consequence; however, a large number of findings were reported which indicated other possible causes for chest pain, such as infection, pleurisy, and hiatus hernia. Incidental CT findings on CTCA have been investigated previously with one large series reporting that 56% of patients had reportable incidental findings [22]. There is continued debate over whether these findings should be reported or followed up [23]. In our study, there appeared to be clear benefits from reporting and following up these incidental findings in terms of discovery of alternative explanations for chest pain and treating them.

This study was a retrospective single arm audit, which entered all patients with stable chest pain into a calcium scoring protocol. The limitations of the study are that (a) we could not compare calcium scoring with CTCA to calcium scoring alone and that (b) we made an assumption that patients with a zero calcium score and low pretest probability required no further cardiac investigations unless they had a recurrence of chest pain.

## 5. Conclusions

The study demonstrates the utility of calcium scoring as a sole CT examination in patients presenting with chest pain. Combined with pretest probability using a standard risk table, all cases of moderate and significant coronary disease demonstrated on subsequent angiography were excluded. A significant number of patients could therefore be reassured and discharged from further investigations based on these two measures. Calcium scoring alone may lead to missing some soft plaque lesions, which may be of significance. This methodology appears to reduce the need for subsequent investigations but does not eliminate it. The study also demonstrated that examination of the associated CT scans did reveal a number of noncardiac findings, which may have either caused or contributed to chest symptoms.

## Figures and Tables

**Table 1:** The relationship between calcium scores and diabetes and hypertension.

Sex	Diabetes	Hypertension		*N*	Mean	Std. deviation	Std. error mean
Female	No	No	Calcium score *P* = NS	157	80.661	243.6941	19.4489
		Yes	Calcium score *P* = NS	70	117.361	238.9071	28.5549
	Yes	No	Calcium score *P* = .043	17	33.024	61.9628	15.0282
		Yes	Calcium score *P* = .185	15	140.933	391.3516	101.0465

Male	No	No	Calcium score *P* = NS	112	127.568	244.7537	23.1271
		Yes	Calcium score *P* = NS	64	251.000	424.6471	53.0809
	Yes	No	Calcium score *P* = .154	5	412.720	526.3036	235.3701
		Yes	Calcium score *P* = .012	22	297.895	505.1356	107.6953

This table shows the distribution of calcium score in the study population and the relationship to diabetes and hypertension, two major risk factors for coronary disease. The relationship between calcium score and these risk factors is weak. It is strongest for the male gender and diabetes combined with hypertension.

**Table 2:** Calcium score versus pretest risk.

Calcium score	No = low pretest risk	*N* = medium pretest risk	*N* = high pretest risk
Zero calcium score, male	18	20	21
Zero calcium score, female	114	52	29
Calcium score 1–400, male	11	24	76
Calcium score 1–400, female	15	15	17
Calcium score >400, male	1	2	30
Calcium score >400, female	3	5	9

This table shows the numbers of patients with a particular calcium score range and their pretest risk, which is classified as low, medium, or high. The majority of patients with a zero calcium score have a low pretest risk, but significant numbers of patients have a zero calcium score with a high pretest risk.

**Table 3:** Angiogram severity versus pretest risk.

Pretest Risk	Normal angiogram %	Plaque but no significant stenosis (mild) %	Moderate soft plaque stenosis, less than 60% (moderate) %	Severe obstructive disease (severe) %
Low, male	0%	7%	0%	0%
Low, female	2%	3%	0%	0%
Medium, male	0%	0%	0%	0%
Medium, female	0%	2%	0%	5%
High, male	13%	10%	7%	25%
High, female	10%	5%	8%	3%
Total	**25%**	**27%**	**15%**	**33%**

Table showing that 25% of the angiograms performed were normal and 27% showed nonsignificant plaque. 28% of significantly abnormal angiograms had a high pretest risk of coronary disease, but in the high-risk group there were also 23% of normal angiograms. No patient with a low pretest risk had a significantly abnormal angiogram.

**Table 4:** Patient outcomes versus calcium score.

	Discharge	Medical Rx	CABG	PCI	Other investigations/outcomes
Zero calcium score, male, *n* = 59	50	4	0	0	5
Zero calcium score, female, *n* = 195	156	15	0	0	24
Calcium score 1–400, male, *n* = 111	45	20	1	5	40
Calcium score 1–400, female, *n* = 47	12	17	2	2	14
Calcium score >400, male, *n* = 33	3	20	3	4	3
Calcium score >400, female, *n* = 17	1	12	1	1	2
Total	**267**	**88**	**7**	**12**	**88**

Table demonstrating the outcomes of the patient group in relation to their calcium score. The majority of patients with a zero calcium score were discharged unless they had a high pretest risk of coronary disease. No patient with a zero calcium score required revascularization.
